# Transforming Growth Factor Beta Receptor 1 Is Increased following Abstinence from Cocaine Self-Administration, but Not Cocaine Sensitization

**DOI:** 10.1371/journal.pone.0083834

**Published:** 2013-12-30

**Authors:** Amy M. Gancarz-Kausch, Gabrielle L. Schroeder, Clarisse Panganiban, Danielle Adank, Monica S. Humby, Michael A. Kausch, Stewart D. Clark, David M. Dietz

**Affiliations:** 1 Department of Pharmacology & Toxicology, University at Buffalo, Buffalo, New York, United States of America; 2 Research Institute on Addictions, University at Buffalo, Buffalo, New York, United States of America; University of Colorado, United States of America

## Abstract

The addicted phenotype is characterized as a long-lasting, chronically relapsing disorder that persists following long periods of abstinence, suggesting that the underlying molecular changes are stable and endure for long periods even in the absence of drug. Here, we investigated Transforming Growth Factor-Beta Type I receptor (TGF-β R1) expression in the nucleus accumbens (NAc) following periods of withdrawal from cocaine self-administration (SA) and a sensitizing regimen of non-contingent cocaine. Rats were exposed to either (i) repeated systemic injections (cocaine or saline), or (ii) self-administration (cocaine or saline) and underwent a period of forced abstinence (either 1 or 7 days of drug cessation). Withdrawal from cocaine self-administration resulted in an increase in TGF-β R1 protein expression in the NAc compared to saline controls. This increase was specific for volitional cocaine intake as no change in expression was observed following a sensitizing regimen of experimenter-administered cocaine. These findings implicate TGF-β signaling as a novel potential therapeutic target for treating drug addiction.

## Introduction

Drug addiction is a chronic disorder represented by persistent drug-seeking and reoccurring episodes of relapse [1]. Psychomotor stimulant abuse and addiction leads to large economic and societal burdens, yet to date, there is no effective intervention. While recent reports have shed a great deal of insight into the neurobiology of addiction, a more complete understanding of how drug abuse leads to long-term behavioral, cellular, and morphological plasticity is desperately needed in order to establish a treatment for this disabling disease [1]–[6]. The neuroadaptions that are initiated following drug exposure and that remain stable over periods of drug abstinence are of particular interest, as these changes occur in the absence of the drug itself, and may confer a neurobiological mechanism that leads to long-term behavioral changes such as craving and relapse [7].

Time-dependent adaptations in synaptic connectivity, glutamatergic and dopaminergic receptor expression and signaling, and neurotrophic levels have been reported following cessation of cocaine treatment [8]–[17]. Transforming Growth Factor Beta (TGF-β) is a signaling cascade that may be a prospective facilitator of these long-term changes in drug-induced plasticity. TGF-β signaling cascades are widely distributed throughout the central nervous system and have a variety of cellular functions in the adult organism, including apoptosis, cellular homeostasis and tissue repair [18]. The binding of TGF-β to the TGF-β Type I Receptor (TGF-β R1) initiates signal propagation through two mechanisms: a canonical mechanism via SMAD proteins, and a non-canonical SMAD-independent mechanism via activation of extracellular signal-related kinases (ERKs), and signaling cascades associated with actin dynamics such as GTPases [18].

TGF-β R1 and downstream signaling cascades have been implicated in numerous psychiatric disorders, including diseases that are largely comorbid with addiction, such as depression and anxiety [19]–[22]. Moreover, TGF-β has been shown to have a role in mediating adult neurogenesis, a neural mechanism shown to be involved in mediating drug-taking and relapse [23]–[25]. The involvement of TGF-β signaling in mediating neural plasticity marks this pathway as a potential regulator of cellular changes in response to drug taking. To this end, we have investigated changes in TGF-β signaling using two models of drug exposure over varying periods of drug abstinence.

## Methods

### Subjects

Sprague Dawley rats weighing between 300–400 g at the time of testing were used in the experiments. All rats were undisturbed for two days upon arrival to the colony room to allow for habituation, and housed on a 12 hr light-dark cycle with *ad libitum* access to food and water. Rats were housed two per cage for the experimenter-administered cocaine experiments. For the self-administration (SA) experiments, rats were singly housed following surgery and for the duration of the experiment in order to protect the catheter/harness assembly. Testing took place seven days/week during the rats’ dark phase of the light-dark cycle. This study was conducted in accordance with the guidelines set up by the Institutional Animal Care and Use Committee of the State University of New York at Buffalo.

### Locomotor Apparatus

Locomotor activity was recorded by an infrared motion-sensor system (AccuScan Instruments) fitted outside plastic cages (40×40×30 cm) containing a thin layer of corncob bedding that were cleaned between each test session. The Fusion activity-monitoring system software monitors infrared beam breaks at a frequency of 0.01 sec. The interruption of any beam not interrupted during the previous sample was interpreted as an activity score.

### Self-administration Test Chambers

Sixteen standard experimental test chambers (MED Associates, Inc.) equipped with two snout-poke holes located on one wall of the test chamber monitored with infrared detectors were used for SA experiments. Two stimulus lights were mounted above the snout-poke holes, and a houselight was mounted in the middle of the back wall of the test chamber. All test chambers were housed in sound attenuating chambers, which mitigate all external light sources and sounds, including sounds from the syringe infusion pumps. Test chambers were computer controlled through a MED Associates interface with MED-PC with a temporal resolution of 0.01 sec.

### Drug

(−)-Cocaine hydrochloride (gifted by NIDA) was dissolved in sterile 0.9% saline. For the experimenter-administered cocaine experiment, a constant injection volume of 1.0 ml/kg was used. Saline or 10.0 mg/kg cocaine was injected intraperitoneally (i.p.) immediately prior to the start of each session in the home cage or in locomotor chamber. For SA experiments, cocaine solutions (4.5 mg/ml) made on a weekly basis were delivered via a syringe pump. Pump durations were adjusted according to body weight on a daily basis in order to deliver the correct dose of drug (1.0 mg/kg/infusion cocaine).

### Jugular Catheterization and Patency Test

Rats were implanted with chronic indwelling jugular catheters and allowed 7 days to recover following surgery as previously described [26], [27]. Catheters were flushed daily with 0.2 ml solution of enrofloxacin (4 mg/ml) mixed in a heparinized saline solution (50 IU/ml in 0.9% sterile saline) to preserve catheter patency. At the end of behavioral testing, each animal received an intravenous (IV) infusion of ketamine hydrochloride (0.5 mg/kg in 0.05 ml saline) and the behavioral response was observed to verify catheter patency. Loss of muscle tone and righting reflexes served as behavioral indicators of patency. Only rats with patent catheters were used in data analysis.

### Self-administration

One week after jugular catheter surgery, the rats were assigned to self-administer either 1.0 mg/kg/inf cocaine or saline. Rats were tested for SA over 10 test sessions, during which responses to the active snout-poke resulted in IV injections of cocaine (or saline) according to a Fixed Ratio 1 (FR1) schedule of reinforcement followed by a 30 sec time-out period. Infusions were accompanied by a 5 sec illumination of the stimulus light above the active snout-poke hole and the houselight was extinguished for the duration of the time-out period. Snout-poke responses to the inactive alternative resulted in no programmed consequences. Session durations were terminated after either a 2-hr duration or 20 infusions had been earned (cumulative dose 20 mg/kg), whichever occurred first. Following testing, catheters were flushed and rats were returned to the colony room. The criterion for acquisition of cocaine SA was an average of 10 infusions per day during the 10-session cocaine test phase.

### Withdrawal

Following SA, rats were counterbalanced according to SA performance and assigned to one of two withdrawal time points (1 or 7 days). In the 1-day withdrawal group (cocaine, n = 7; saline, n = 6), brains were harvested 24 hrs after the last day of SA testing. Rats were sacrificed by rapid decapitation, brains were removed and sliced into 1 mm thick sections using a brain matrix (Braintree Scientific), and 2 mm diameter tissue punches from the nucleus accumbens (NAc) were collected and rapidly frozen on dry ice. In the 7-day withdrawal group (cocaine, n = 8; saline, n = 7), rats were returned to their home cages in the colony room and left undisturbed for one week, following which brains were removed and NAc tissue punches were collected in an identical manner.

### Locomotor Response to Experimenter-administered Cocaine

In order to control for any basal differences in motor responses, rats were tested for locomotor response to novelty (1 hr duration using the Accuscan Monitoring system). Rats were then counterbalanced according to the locomotor scores (data not shown) and were assigned to receive seven daily i.p. injections of either 10 mg/kg cocaine or saline. Injections occurred in the test room on days 1, 3, 5 & 7 and animals were placed in the locomotor chambers for 1 hr. Injections on days 2, 4 & 6 occurred in the home cage [28], [29]. Following the last day of injections, rats were returned to the colony room and remained undisturbed in their home cages. In the 1-day withdrawal group (cocaine, n = 7; saline, n = 8), brains were removed and NAc tissue punches collected 24 hrs after the last injection using the same procedures as described for the SA experiments. In the 7-day withdrawal group (cocaine, n = 10; saline, n = 10), rats were left undisturbed in the colony room for one week, following which brains were removed and NAc tissue punches were collected in an identical manner.

### Western Blot Quantification of TGF-β R1

Protein expression levels of TGF-β R1 were analyzed by Western blotting as previously described [30], [31]. Briefly, frozen NAc tissue punches from each rat were homogenized in 30 μl of homogenization buffer containing 320 mM sucrose, 5 mM HEPES buffer, 1% SDS, phosphatase inhibitor cocktails I and II (Sigma), and protease inhibitors (Roche). Protein concentrations were determined, and a total of 30 μg of protein was loaded onto 10% Tris-SDS polyacrylamide gels for electrophoresis fractionation. Proteins were transferred to nitrocellulose membranes, blocked with 5% non-fat milk, and incubated overnight at 4°C with primary antibodies (anti-rabbit TGF-β Receptor I, Cell Signaling, 1∶500; anti-mouse β-actin, Cell Signaling, 1∶10,000) in Odyssey blocking buffer. After thorough washing with 0.1% Tween-20 in phosphate-buffered saline, membranes were incubated with IRDye secondary antibodies (1∶5000; Li-Cor) dissolved in Odyssey blocking buffer for 1 hr at room temperature. The blots were imaged with the Odyssey Infrared Imaging system (Li-Cor) and quantified by densitometry using NIH Image J. The amount of protein blotted onto each lane was normalized to levels of β-actin.

### Data Analysis

Locomotor activity during the experimenter-administered cocaine experiment, and performance during SA were analyzed using a two-factor within-subject Analyses of Variance (ANOVAs) with the between-session variable as drug group (cocaine/saline) and the within-subject variable as time (day of injections or sessions, respectively). The primary dependent measure of locomotor activity used for statistical analysis was the total number of beam breaks, and the primary dependent measure for SA acquisition was the number of infusions. Western blot data was compared using two-tailed Student’s t-tests. Statistical significance was set at *p*<0.05 using SPSS statistical software. All data are represented as the mean ± SEM.

## Results

**Figure 1:**
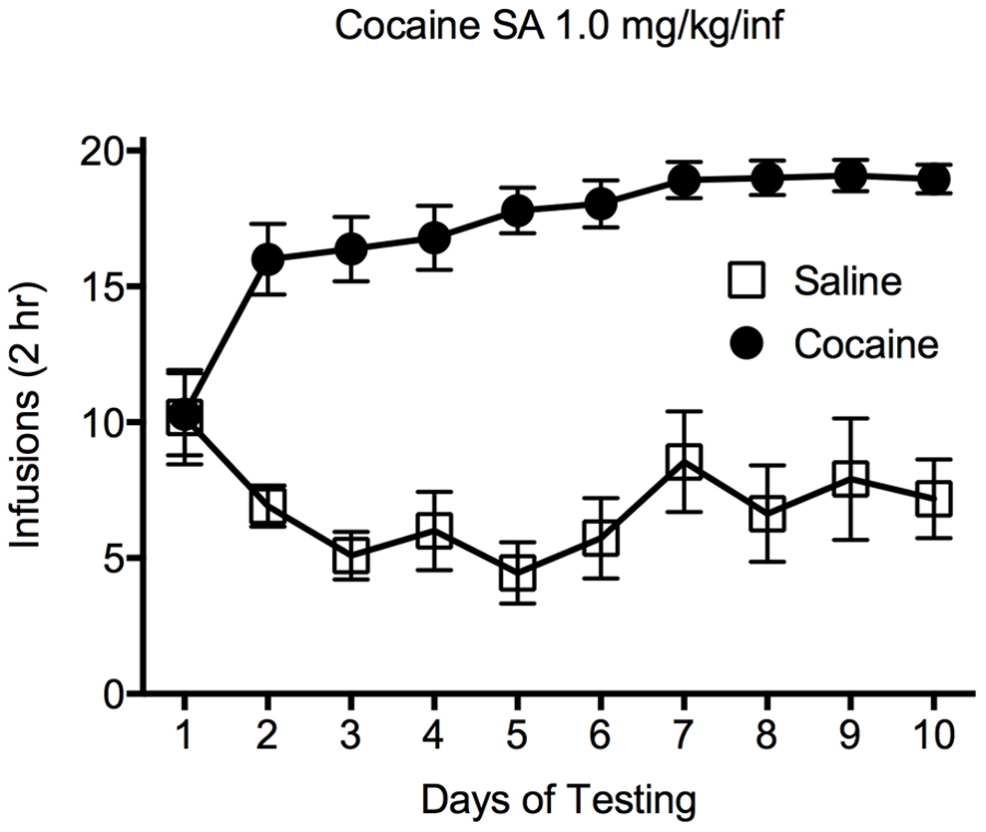
Self-administration of cocaine or saline. This plot shows the number of infusions earned across ten days of self-administration of cocaine (1.0 mg/kg/inf) or saline. Closed circles represent animals self-administering cocaine, open squares indicate animals receiving infusions of saline. Data are expressed as the average number of infusions (± SEM) over the ten days of cocaine/saline self-administration; *p<0.05.

Analysis of the results for the SA experiments showed that there was a main effect of drug (cocaine/saline) [F(1,250) = 617.6; *p*<0.0001] and a significant interaction between cocaine and saline SA across the 10 days of testing [F(9,250) = 9.297; *p*<0.001]. Follow-up post hoc (Tukey’s) tests revealed that rats responding for infusions of cocaine had a significantly greater number of infusions than animals responding for saline on sessions 2–10, indicating that rats acquired cocaine self-administration (**Figure 1**).

**Figure 2:**
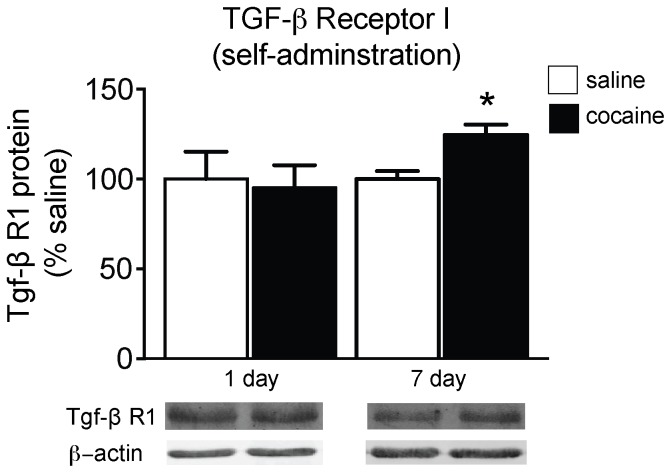
TGF-β R1 expression following active cocaine exposure. Relative TGF-β R1 protein expression in the NAC of rats following 1 or 7 days of withdrawal from cocaine (1.0 mg/kg/inf; 10 days) or saline self-administration; *p<0.05 compared to saline.

To examine if TGF-β receptor expression is regulated following cocaine SA, protein levels of TGF-β R1 from whole cell lysates of NAc tissue punches from rats with a history of cocaine and following a period of 1 or 7 days of withdrawal were analyzed. TGF-β R1 expression was unchanged following 1 day of withdrawal from cocaine SA compared to the saline group (**Figure 2**). In comparison, there was a significant increase in TGF-β R1 expression in the NAc of animals with a history of cocaine SA following a 7-day withdrawal period compared to saline controls [*t*(13) = 3.269; *p*<0.001], indicating that TGF-β R1 expression is up-regulated following a period of drug cessation in a time-dependent manner (**Figure 2**).

**Figure 3:**
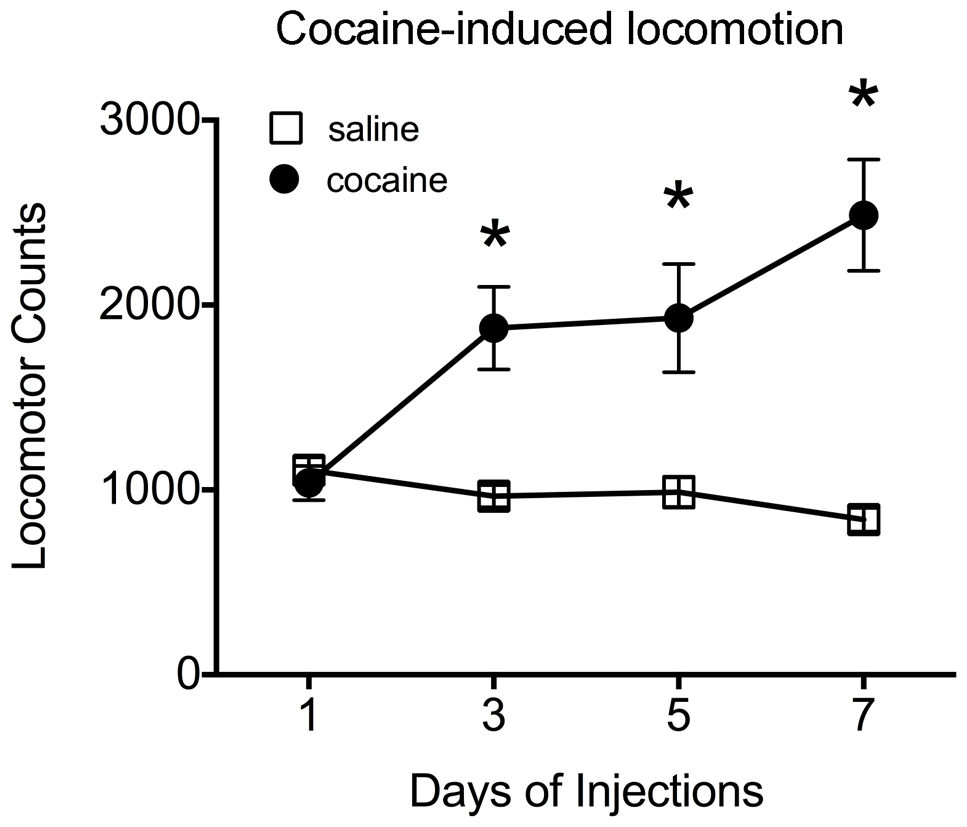
Locomotor response to a sensitizing regimen of cocaine. This plot shows locomotor activity in response to repeated injections of cocaine (10 mg/kg, i.p.) or saline. Closed circles represent animals receiving injections of cocaine; open squares indicate animals receiving injections of saline. Data are shown as group averages (± SEM); *p<0.05 compared to saline.

We next asked if this increase in TGF-β R1 expression occurred following all cocaine regimens, and thus simply a result of drug exposure. To this end, we used a regimen of cocaine known to induce behavioral sensitization, which thought to have, at least in part, common neural substrates that underlie addiction [32]. As shown in **Figure 3**, animals receiving repeated systemic injections of cocaine exhibited an increase in locomotor activity over time, suggesting the development of behavioral sensitization. There was a significant interaction between group (cocaine/saline) and day [F(4,120) = 7.718; *p*<0.01], and a main effect of drug group (cocaine/saline) [F(1,30) = 19.01; *p*<0.001]. Post-hoc tests showed that rats in the cocaine group had significantly greater locomotor activity on days 2–4 of injections compared to day 1 and compared to saline (all *p*’s <0.05), whereas no differences in locomotor activity were observed across time in animals injected with saline. Furthermore, post-hoc tests showed that animals injected with cocaine exhibited significantly more locomotor activity compared to rats receiving injections of saline at Days 2–4 of testing.

**Figure 4:**
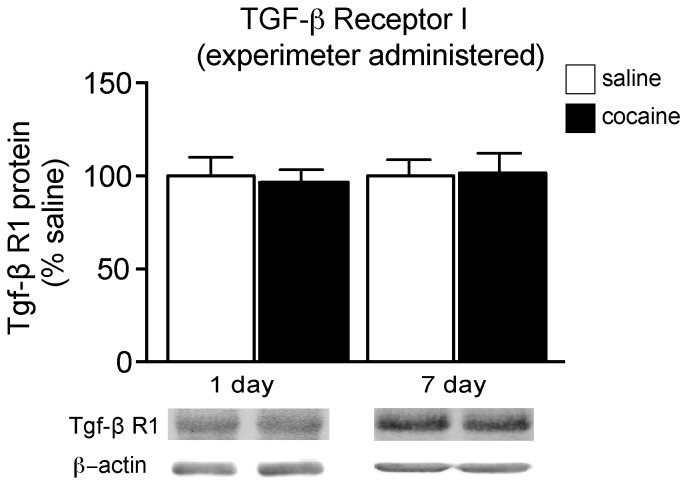
TGF-β R1 expression following experimenter-administered cocaine. Relative TGF-β R1 protein expression in the NAc of rats following 1 or 7 days of withdrawal from a sensitizing regimen of cocaine (10 mg/kg; i.p, 7 days) or saline; *p<0.05 compared to saline.

Levels of TGF-β R1 expression were examined following experimenter-administered cocaine, and surprisingly, we found a different expression pattern to that observed following SA. Twenty-four hours following experimenter administered cocaine, we found no change in TGF-β receptor protein expression compared to saline treated [t(18) = 0.9140, p>0.05], which remained unchanged after a 7-day withdrawal period [t(13) = 0.2850, p>0.05] (**Figure 4**). Taken together, these data indicate TGF-β R1 signaling is regulated only following withdrawal from cocaine SA, but not experimenter-administered cocaine.

## Discussion

The results of this study identify the TGF-β receptor as a previously unknown molecular adaption following periods of cocaine cessation. The time-dependent regulation of TGF-β R1 occurred following active but not passive cocaine exposure, as the increase in protein expression was observed only after withdrawal from cocaine SA, and not after withdrawal from a sensitizing regimen of cocaine.

It is intriguing to speculate about the involvement of the TGF-β receptor-signaling cascade following a history of cocaine self-administration. TGF-β signaling pathways may potentially regulate actin cycling directly to alter structural changes in the NAc. Repeated exposure to psychomotor stimulants, such as cocaine, results in morphological changes to NAc medium spiny neurons (MSNs) including increases in dendritic spine density [30], [33]–[35]. One mechanism that directly links TGF-β signaling to cocaine-induced morphological plasticity is through a documented ability of TGF-β to alter the actin cytoarchitecture through Rho GTPase signaling, which has previously been linked to cocaine-induced structural plasticity [30], [36]–[39]. Moreover, drug-induced structural plasticity of dendritic spine morphology exists along a continuum that appears to be a function of time from cessation of drug exposure, method of intake, drug paradigm and re-exposure to cues previously associated with drug availability [2], [40]–[44]. Further studies are needed to identify the exact role of TGF-β receptor signaling in mediating cocaine-induced structural changes, and how such alterations may impact relapse behaviors.

Our findings demonstrate that TGF-β receptor expression is increased only in the NAc of animals that self-administer cocaine and not following experimenter-administered drug. There are numerous examples of neurobiological changes that occur differentially following active versus passive drug exposure [45]–[51] and these changes may underlie distinct behavioral changes such as drug sensitivity [52], [53]. It is important to note that several procedural differences between the SA and experimenter-administered cocaine protocols, such as dosing regimens and pharmacokinetics, may have a role in the selective increase in TGF-β R1 expression following SA and future studies are needed to examine the contributions of these factors.

It is worth noting that our findings differ from those of Maze *et al.* [54], in which the authors report an increase in TGF-β R1 following experimenter-administered cocaine. However, there are significant procedural differences between the Maze *et al.* study and the current experiments that may account for such paradoxical results: (i) Maze *et al.* tested mice, rather than rats as used in the current experiments; (ii) mRNA were measured in the Maze study, whereas we report protein expression.

While future studies are needed to develop a more complete temporal profile of TGF-β receptor expression following cocaine SA and withdrawal (i.e., prolonged periods of forced abstinence), our results demonstrate that the TGF-β Type I receptor is a potential target for intervention towards an effective pharmacotherapy in treating addiction. Further studies will determine how cocaine mediates down-stream signaling of TGF-β and how such cascades result in long-term cellular, morphological, and behavioral plasticity.
